# Congenital tuberculosis in a neonate following *in vitro* fertilization-embryo transfer: A case report

**DOI:** 10.3389/fped.2022.985707

**Published:** 2022-08-11

**Authors:** Guiying Zhuang, Linzhi Yang, Liuhong Qu, Weiqi Liu, Huanjin Zhu

**Affiliations:** ^1^Division of Neonatology, The Maternal and Children Health Care Hospital (Huzhong Hospital) of Huadu, Guangzhou, China; ^2^Department of Clinical Laboratory, The Maternal and Children Health Care Hospital (Huzhong Hospital) of Huadu, Guangzhou, China

**Keywords:** congenital tuberculosis, *in vitro* fertilization-embryo transfer, infection, *Mycobacterium tuberculosis*, neonate

## Abstract

**Background:**

Congenital tuberculosis is becoming increasingly common, but congenital tuberculosis infection in neonates following *in vitro* fertilization and embryo transfer (IVF-ET) has been rarely reported; a diagnosis of congenital tuberculosis is often delayed due to the non-specificity of maternal IVF treatments and clinical manifestations during pregnancy—particularly in low-birth-weight preterm infants.

**Case presentation:**

We herein report a case of congenital tuberculosis. The infant was born at 27+5 weeks of gestation and was admitted to the hospital due to hypopnea after birth. Due to a poor response to treatment, we conducted pathogenic microorganism metagenomic analysis to assess the nucleotide sequences within the *Mycobacterium tuberculosis* complex. After collecting sputum, the strains from the tuberculosis analysis were isolated and confirmed. From a detailed examination of the mother and in accordance with the child's congenital tuberculosis, we confirmed the diagnosis of pelvic tuberculosis.

**Conclusion:**

IVF treatment and pregnancy can exacerbate latent tuberculosis, especially in women from a family with a history of tuberculosis infections. We posit that the optimal way to prevent neonatal congenital tuberculosis in IVF-ET is to procure a detailed maternal medical or family history and to identify and treat maternal tuberculosis during IVF treatment.

## Introduction

Tuberculosis is a disease caused by *Mycobacterium tuberculosis* (*M. tuberculosis*) infection, with an estimated 1.7 billion people (23%) infected worldwide; moreover, there are over 10 million new cases annually (1). Tuberculosis has now surpassed human immunodeficiency virus/acquired immunodeficiency syndrome as the major cause of death caused by infectious pathogens (2). Congenital tuberculosis may be caused by inhalation or ingestion of *M. tuberculosis* by the mother and then passing the bacterium through the placenta into the fetus, or by the fetus passing through the birth canal. Peng et al. (3) studied 170 children with congenital tuberculosis and found that most had no specific manifestations for the first 2–3 weeks and that the majority of mothers were diagnosed following the child's diagnosis. Such an early clinical-stage diagnosis thus engenders greater demands: due to the atypical early clinical manifestations, the mortality rate was reported to be as high as 44% (4). Although the development of *in vitro* fertilization (IVF) and other assisted reproductive technologies (ARTs) over recent decades has allowed an increasing number of infertile women to conceive healthy babies, physical examination of the mother's own health is often ignored during the IVF treatment process, and this may conceal disease in the offspring. Among the existing publications on congenital tuberculosis, there are few reports of congenital tuberculosis infection in infants born from IVF. In this report, we describe a case of congenital tuberculosis in a premature infant born from IVF/ART and confirmed by macromicro (DNA) testing and positive acid-fast bacilli in sputum smears.

## Case report

### The neonate

**Figure 1:**
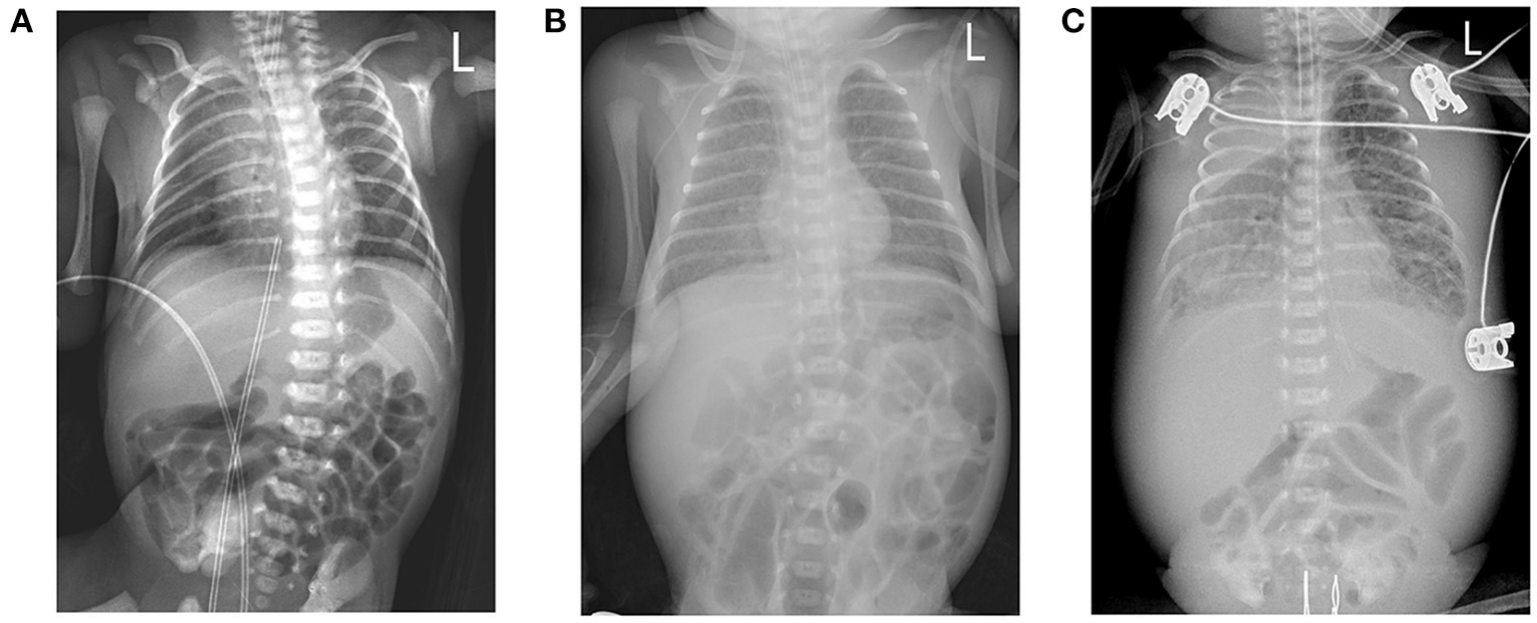
**(A)** Chest X-ray image shows a bilateral thickening of lung markings, with patchy and blurred shadows in the lower lung fields. **(B)** Chest X-ray image depicts thickening and blurring of bilateral lung markings, with small patches of fuzzy shadows in the lung fields. **(C)** Chest X-ray image shows that the texture of both lungs was thickened, increased, and blurred. Large dense shadows of uniform density were observed in the right upper lung field, and small patchy blurred shadows were found in the remaining lung fields. There was a narrow band of increased density in the field of the right lower lung that contained a clear boundary.

A Chinese mother delivered a male baby by cesarean section at 27+5 weeks after IVF-embryo transfer (IVF-ET). The neonate's birthweight was 1,060 g, and his Apgar scores were 9-10-10. After birth, the boy was admitted to the NICU due to shallow breathing. Physical examination upon admission disclosed a body temperature of 36°C, a pulse rate of 144 beats/min, respiratory rate of 42 beats/min; and blood pressure of 66/44 mmHg. The child exhibited shallow breathing, grunting, and low breath sounds in both lungs; cardiac and abdominal examination, however, showed no abnormal signs. Initial examination at the time of hospitalization showed the following: blood test results disclosed white blood cells (WBCs) at 17.58 × 10^9^/L, neutrophils (NEU) at 42.50%, hemoglobin (HGB) at 155 g/L, platelets (Plt) at 208 × 10^9^/L; a C-reactive protein (CRP) level < 0.5 mg/L; and a procalcitonin (PCT) of 0.41 ng/mL. Chest X-ray displayed lung changes and neonatal pneumonia in the premature infant, but plain abdominal X-ray revealed no abnormalities (Figure 1A). After admission to the hospital, the child was mechanically ventilated and treated with pulmonary surfactant (PS) and intravenous nutrition.

Mechanical ventilation was changed on the second day after hospitalization to non-invasive assisted ventilation, and we noted no apnea or periodic breathing under continuous non-invasive assisted ventilation over the next 14 days; transcutaneous oxygen saturation (TcSO_2_) was maintained at 90–94%. During this period the child was breastfed and occasionally experienced abdominal distension, vomiting, and feeding intolerance. With a maximal feeding amount of 30 ml/Kg, body weight increased to 1,200 g, and the child's growth curve was at the 25^th^ percentile.

However, the patient's condition changed 15 days after birth. In the non-invasive assisted ventilation (nCPAP) mode, the child manifested clinical symptoms such as dyspnea, obvious abdominal distension, and weakened bowel sounds. Thus, the boy's condition changed 2 weeks after being born prematurely; and although nosocomial infection was considered, the infection-related examination showed obvious abnormalities in his complete blood count (WBCs, 12.11 × 10^9^/L; neutrophils, 72.9%; HGB, 106 g/L; PLTs, 247 × 10^9^/L; a CRP of 2.7 mg/L; and a PCT of 0.54 ng/ml). However, blood cultures from two sites as well as sputum, stool, and urine culture did not divulge any bacterial growth. Chest X-ray displayed a thickening of lung markings and small flakes of fuzzy shadows, supporting a diagnosis of neonatal pneumonia. The abdominal intestine was also slightly distended, and intestinal dysfunction was considered (Figure 1B). We therefore administered mechanical ventilation and advised fasting, gastrointestinal decompression, and anti-infective and other treatments.

On day 18 after birth and under continuous mechanical ventilation, the boy's airway secretions increased, his sputum became thick, and erythematous, papulomacular, coalescent rash with indistinct borders (not consistent with common beningn neonatal skin rashes or drug rashes). The blood test results showed 15.63 × 10^9^/L WBCs, 81.3% NEUs, an HGB of 111 g/L, Plt of 144 × 10^9^/L, CRP of 95.1 mg/L, and a PCT of 6.46 ng/mL. Thus, compared with his previous test results, NEUs, CRP, and PCT increased, while PLT decreased. Sputum culture showed the growth of ESBL-producing *Klebsiella pneumoniae* subsp., as fungal (1-3)-β-D-glucopyranose was 290.71 pg/mL (the normal reference interval is 0–70 pg/mL); B-ultrasonography revealed a small amount of fluid in the intestinal space. Chest X-ray examination displayed progressive aggravation of pneumonia, and the abdominal portion was slightly thickened and stiff on *X*-ray. According to the examination results, we recognized that the child possessed a serious infection, and meropenem and fluconazole were administered as anti-infectives.

**Figure 2:**
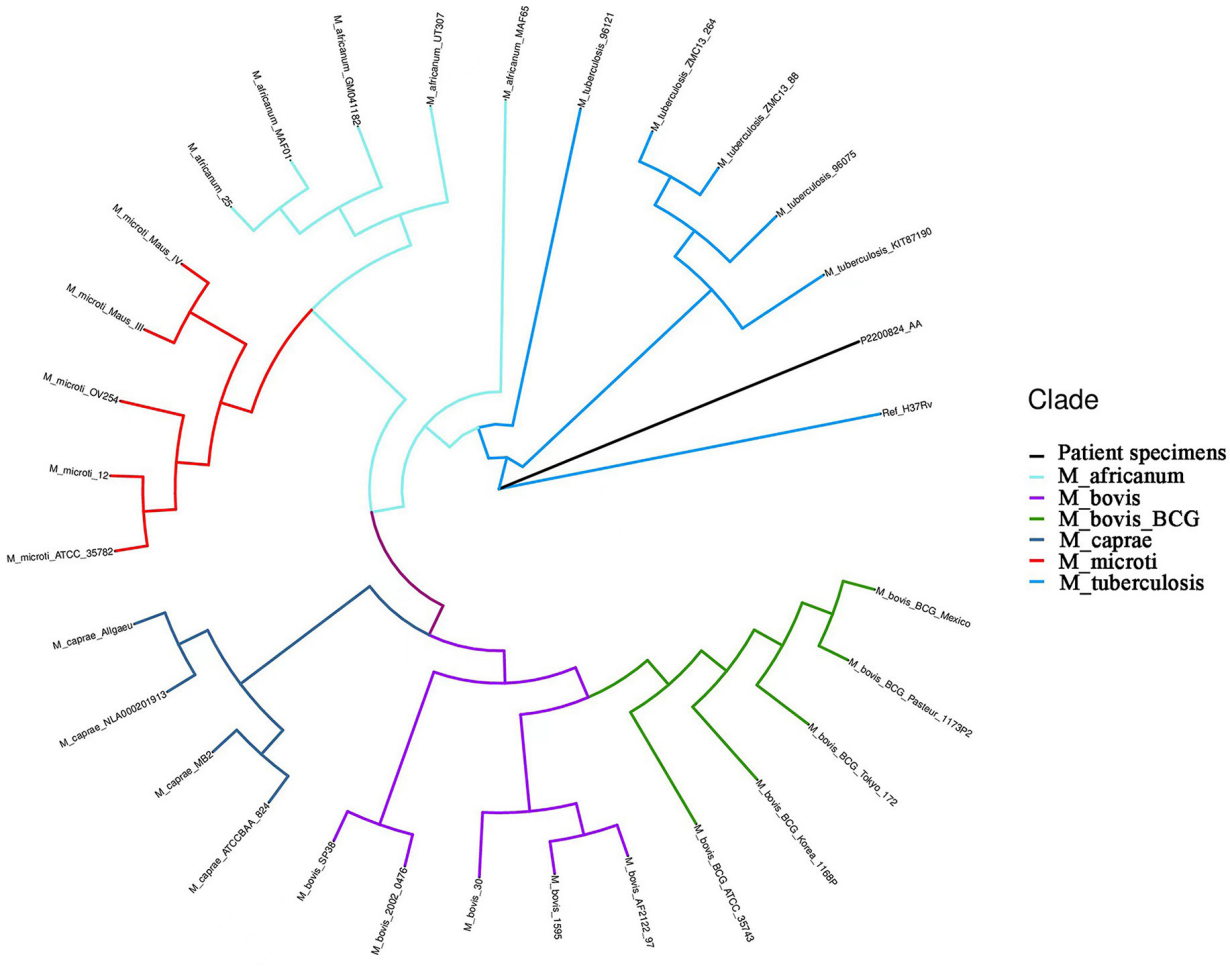
Hierarchical clustering dendrogram of the growth detection of the serum metagene *Mycobacterium tuberculosis* complex. Ref_H37Rv: reference of *M.tuberculosis* H37Rv from NCBI; M_africanum: *Mycobacterium africanum*; M_bovis: *Mycobacterium bovis*; M_bovis_BCG: *Mycobacterium bovis* bacillus Calmette-Guérin (BCG); M_caprae: *Mycobacterium caprine*; M_microti: *Mycobacterium microti*; M_tuberculosis: *M.tuberculosis*; P2200824_AA: patient specimens.

**Figure 3:**
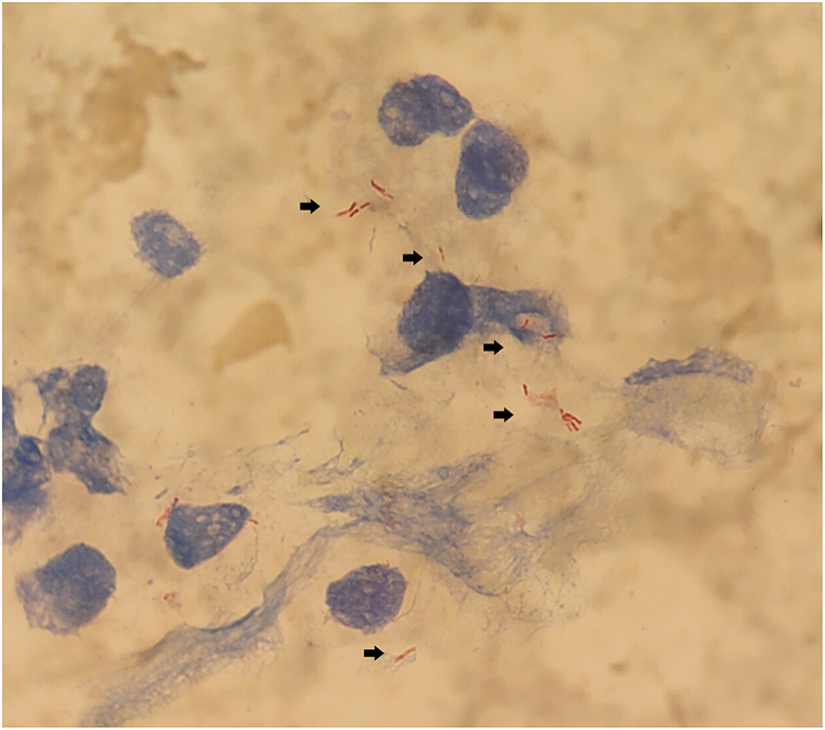
Acid-fast bacilli are shown in the neonatal sputum.

The boy's condition further deteriorated from the 19^th^ to 22^nd^ days after birth. During this period, fever occurred for the first time; and even under continuous mechanical ventilation, his complexion remained cyanotic, and his TcSO_2_ was difficult to maintain at 90%. The difference in percutaneous oxygen saturation between the right upper extremity ductus arteriosus and the right lower extremity ductus arteriosus is larger than 10%. The partial pressure of oxygen (PO_2_) values from the blood gas analysis results on days 19–22 were 37.4 mmHg, 40.3 mmHg, 28.0 mmHg, and 34.6 mmHg, respectively, indicating that the child was hypoxemic. On day 22, blood test results showed that Plts decreased to 28 × 10^9^/L and CRP increased to 134.8 mg/L. Chest X-ray examination on the same day showed large, dense opacities in the right upper lung field; small patchy opaque shadows in both lung fields; an air bronchogram sign; and a small amount of pleural effusion on the right side. We also noted bowel dilatation in the abdomen, and that the intestinal septum was slightly thickened, the left abdominal bowel was stiff, and the necrotizing enterocolitis (NEC) was aggravated (Figure 1C). The ventilator parameters were adjusted upward, nitric oxide and sildenafil were administered, and PS and other treatments were repeated. According to previously reported cases, the aforementioned treatment strategies should have achieved a better treatment effect, but the child's condition became progressively worse, and we investigated whether the condition was complicated by another uncommon pathogenic bacterial infection. We collected the child's serum for pathogenic microorganism metagene analysis, and our results indicated that the number of sequences detected by the growth of the *Mycobacterium tuberculosis* complex was 2,541 (100%) (Figure 2). As a result of the metagene results, the endotracheal aspirate of the child was collected for acid-fast bacilli examination, and the results showed that the endotracheal aspirate was positive for acid-fast bacilli (++) (Figure 3). Therefore, combined with the clinical manifestations and examination results, the child was diagnosed with congenital tuberculosis, and anti-tuberculotic treatment with isoniazid and rifampicin was applied.

The child was, however, found to be in critical condition on day 24 after birth, and due to multiple organ-system dysfunctions and the family's withdrawal of treatment, the child was ultimately declared clinically dead.

### The neonate's parents

The neonate's father was 33 years old at the time of the boy's birth and in good health, and the boy's mother was 35 years old. There was a history of pregnancy, with the mother having undergone IVF-ET procedures and producing twins, and with premature rupture of membranes at 20 weeks of gestation. During the current pregnancy, the mother suffered from connective tissue disease and underwent premature rupture of membranes more than 6 days before delivery; and the child was born with clear amniotic fluid of approximately 30 ml. There had been numerous cases of *M. tuberculosis* infection in the families of both parents, the mother had been infertile for many years; both of her pregnancies were *via* IVF/ET, each resulting in preterm births. The mother complained that although other infectious diseases were investigated during her IVF treatment, tuberculosis was not evaluated; and there was a history of persistent cough 1 month before delivery. We therefore recommend that a mother suspected of having tuberculosis be sent to a higher specialized hospital as soon as possible to complete the relevant examinations. The mother was ultimately diagnosed with pelvic tuberculosis at the Guangzhou Chest Hospital (a superior hospital) and required conventional treatment.

## Discussion

Our current patient was an IVF-ET neonate with congenital tuberculosis, a rare clinical manifestation. The neonate manifested hypopnea at his initial clinical appointment, was not febrile for 14 days after birth, and we noted no abnormality in the inspection results with regard to infection indicators; only the chest X-ray showed lung changes and neonatal pneumonia in this premature infant. Due to the unsatisfactory response to treatment and the worsening severity of his condition, serum metagenomic testing was considered in combination with evaluation for other uncommon pathogenic bacterial infections, and our results revealed *M. tuberculosis* complex. Upon receiving the metagenomic results, sputum was collected to detect acid-fast bacilli to confirm tuberculosis infection, and the mother was ultimately diagnosed with pelvic tuberculosis from the diagnosis of neonatal congenital tuberculosis.

Congenital tuberculosis is relatively rare in clinical practice, particularly in neonates born from IVF-ET. Furthermore, due to different clinical manifestations, misdiagnosis is more likely to occur and result in fatal consequences. While respiratory distress and fever are the principal symptoms of congenital tuberculosis (5, 6), shallow breathing was in our case the initial clinical manifestation, and the condition was relatively stable over the next 14 days. By not noting any fever and by observing normal breastfeeding and significant weight gain of the child, we postulate that clinicians may thereby relax their vigilance regarding the diagnosis and severity of the neonate's disease.

Congenital tuberculosis is thought to be transmitted through the placenta or *via* inhalation of infected amniotic fluid at birth and the ingestion of infected substances (7). Because congenital tuberculosis often requires surgery or an autopsy to determine its mode of transmission, we often do not know the specific transmission route. In the present case, as no autopsy was performed on the child, it remained unclear as to how the neonate was infected with congenital tuberculosis.

When our neonatal patient was diagnosed with congenital tuberculosis, his chest radiograph showed a large dense opacity in the right upper lung field, small patchy fuzzy opacities in both lung fields, and a small amount of pleural effusion on the right side. Serum metagene analysis subsequently revealed the *M. tuberculosis* complex. The sputum of the child was collected as a result of the metagenomic analysis, and the results showed the presence of acid-fast bacilli. Beizke (8) established criteria in 1935 to distinguish between congenital tuberculosis and postnatally acquired tuberculosis, and our case met Beizke's criteria for the diagnosis of congenital tuberculosis; i.e., the isolation of *M. tuberculosis* from the boy.

Previous studies have shown that a majority of mothers with congenital tuberculosis do not possess a previous history of tuberculosis infection (3, 9) and that most mothers are diagnosed with tuberculosis postpartum or when their children are diagnosed with congenital tuberculosis (10). These previous findings are consistent with the final diagnosis of pelvic tuberculosis in the mother in our case after her child was diagnosed with congenital tuberculosis. In the present case, the mother exhibited no history of tuberculosis infection, and only continued to cough for ~1 month prior to delivery; this did not attract the attention of the obstetrician and resulted in the onset of congenital tuberculosis. We therefore suggest that pregnant women who manifest no obvious symptoms of infection during pregnancy, but continue to cough, should remain vigilant; and, if necessary, pregnant women should be evaluated for *M. tuberculosis*.

Infertility over 12 months has been reported to occur earlier in developing countries and ranges from 6.9 to 9.3% (11). In a prospective study in India, the investigators found that the incidence of infertility generated by genital tuberculosis was 3% (12). While IVF has become a relatively common treatment method used for infertile women in recent years, many women are not tested for *M. tuberculosis* before undergoing IVF protocols (10, 13). The application of IVF may thereby emerge as a potential risk factor for congenital tuberculosis (14). Samedi et al. (15) reported a case of congenital tuberculosis following IVF in which the mother exhibited uncontrollable epileptic seizures during preterm birth; these authors isolated *M. tuberculosis* in the maternal placenta, urine, gastric aspirates, and sputum. However, in the present case, the mother displayed a persistent cough only 1 month before delivery; and after the baby was diagnosed with congenital tuberculosis, a detailed examination of the mother revealed her pelvic tuberculosis.

The use of glucocorticoids during maternal preparations for IVF can sensitize the ovaries to gonadotropin stimulation (16, 17), and due to the increased level of estradiol after ovulation induction, both hormones can suppress the immune system and reduce maternal immunoresistance. In addition, changes to maternal hormone concentrations during pregnancy (especially the increase in estrogen) are capable of inhibiting the immune function of maternal lymphocytes (18), thus reducing maternal resistance and thereby precipitating tuberculosis or its recurrence in pregnant women. The appearance of this condition may have been due to the recurrence of pelvic tuberculosis caused by a diminution in maternal resistance during IVF treatment and pregnancy, which ultimately provoked the onset of congenital tuberculosis.

## Data availability statement

The original contributions presented in the study are included in the article/Supplementary materials, further inquiries can be directed to the corresponding author/s.

## Ethics statement

This study was approved by the Ethics Committee of the Maternal and Child Health Hospital of Huadu District (no. 2022-035). Written informed consent to participate in this study was provided by the participants' legal guardian/next of kin. Written informed consent was obtained from the minor(s)' legal guardian/next of kin for the publication of any potentially identifiable images or data included in this article.

## Author contributions

GZ and WL conceived the study. WL drafted the manuscript. LQ revised the manuscript. LY collected patient medical records. HZ supported laboratory data collection. All authors participated in the interpretation of the results and approved the version of the final manuscript.

## Conflict of interest

The authors declare that the research was conducted in the absence of any commercial or financial relationships that could be construed as a potential conflict of interest.

## Publisher's note

All claims expressed in this article are solely those of the authors and do not necessarily represent those of their affiliated organizations, or those of the publisher, the editors and the reviewers. Any product that may be evaluated in this article, or claim that may be made by its manufacturer, is not guaranteed or endorsed by the publisher.

## Abbreviations

CRP: C-reactive protein
HGB: hemoglobin
IVF: *in vitro* fertilization
IVF-ET: *in vitro* fertilization and embryo transfer
*M. tuberculosis*: *Mycobacterium tuberculosis*
NEUs: neutrophils
PCT: procalcitonin
Plts: platelets
TcSO_2_: transcutaneous oxygen saturation
WBCs: white blood cells.
